# Pharmacological Efficacy of Ginseng against Respiratory Tract Infections

**DOI:** 10.3390/molecules26134095

**Published:** 2021-07-05

**Authors:** Abdulrhman Alsayari, Abdullatif Bin Muhsinah, Dalia Almaghaslah, Sivakumar Annadurai, Shadma Wahab

**Affiliations:** 1 Department of Pharmacognosy, College of Pharmacy, King Khalid University, Abha 61421, Saudi Arabia; alsayari@kku.edu.sa (A.A.); ajmohsnah@kku.edu.sa (A.B.M.); sannadurai@kku.edu.sa (S.A.); 2 Department of Clinical Pharmacy, College of Pharmacy, King Khalid University, Abha 61421, Saudi Arabia; damoazle@kku.edu.sa

**Keywords:** ginseng, respiratory tract infection, immuno-modulatory effects, cytokines, antiviral activity, antibacterial activity

## Abstract

Respiratory tract infections are underestimated, as they are mild and generally not incapacitating. In clinical medicine, however, these infections are considered a prevalent problem. By 2030, the third most comprehensive reason for death worldwide will be chronic obstructive pulmonary disease (COPD), according to the World Health Organization. The current arsenal of anti-inflammatory drugs shows little or no benefits against COPD. For thousands of years, herbal drugs have been used to cure numerous illnesses; they exhibit promising results and enhance physical performance. Ginseng is one such herbal medicine, known to alleviate pro-inflammatory chemokines and cytokines (IL-2, IL-4, IFN-γ, TNF-α, IL-5, IL-6, IL-8) formed by macrophages and epithelial cells. Furthermore, the mechanisms of action of ginsenoside are still not fully understood. Various clinical trials of ginseng have exhibited a reduction of repeated colds and the flu. In this review, ginseng’s structural features, the pathogenicity of microbial infections, and the immunomodulatory, antiviral, and anti-bacterial effects of ginseng were discussed. The focus was on the latest animal studies and human clinical trials that corroborate ginseng’s role as a therapy for treating respiratory tract infections. The article concluded with future directions and significant challenges. This review would be a valuable addition to the knowledge base for researchers in understanding the promising role of ginseng in treating respiratory tract infections. Further analysis needs to be re-focused on clinical trials to study ginseng’s efficacy and safety in treating pathogenic infections and in determining ginseng-drug interactions.

## 1. Introduction

According to MedlinePlus, lung disease is considered any problem in the lungs that prevents them from working correctly. The standard classifications of lung diseases are restrictive, obstructive, or vascular. WHO estimates that the third most comprehensive reason for death worldwide by 2030 may be chronic obstructive pulmonary disease (COPD). The problem of respiratory infections is underestimated, as these are generally mild and not incapacitating. The majority of infections are caused by cosmopolitan agents, while geographical or tropical infections are rare.

In clinical medicine, respiratory tract infections (RTIs) are considered prevalent and pose vital problems. Antibiotics are commonly prescribed to treat and manage respiratory infections, even though published literature indicates that they rarely benefit patients. Nasal pharyngitis, acute bronchitis, and non-specific upper respiratory tract infections are caused by respiratory viruses [1]. Several different types of viruses may infect the respiratory tract; these include the adenovirus, rhinovirus, parainfluenza virus, coronavirus, enterovirus, respiratory syncytial virus, and influenza virus. SARS-CoV, SARS-CoV-2, and MERS-CoV are also agents of respiratory infections.

**Figure 1:**
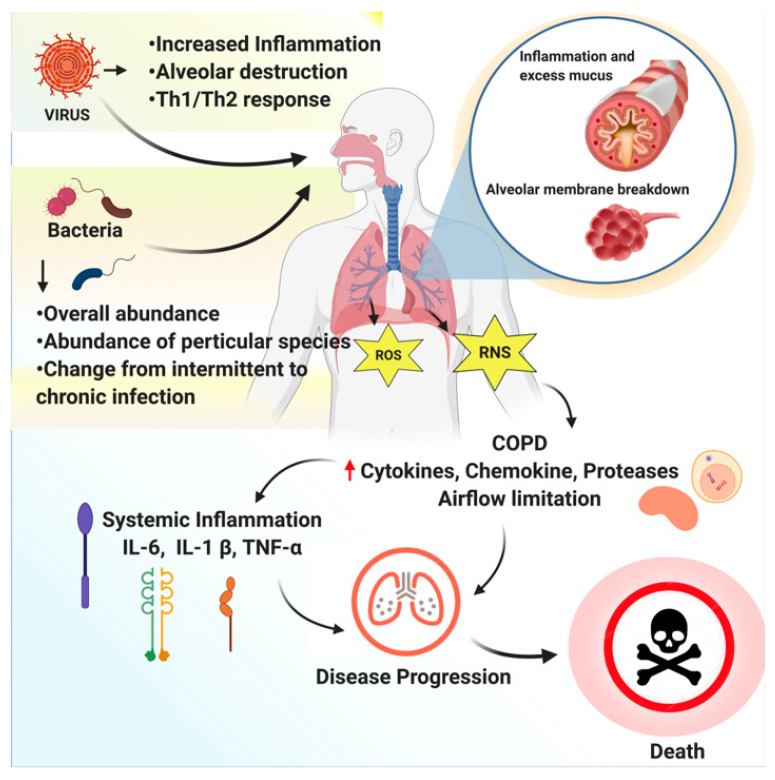
An overview of respiratory tract infections caused by bacteria and viruses. Respiratory pathogens increase the chance of intermittent to chronic lung infection by increasing inflammation and alveolar destruction. Generation of reactive oxygen species (ROS) and reactive nitrogen species (RNS) leads to increase cytokines, chemokines, protease, and limitation of airflow that induce the severity and progression of COPD, systemic inflammation, and lung disease progression and decrease patient survival.

The risk of RTIs is increasing in children and in adults with immune deficiencies due to indoor and outdoor air pollutants which impair their lungs [2,3]. Other causes of the rapid spread of RTIs are the cough and sneeze of an infected person. RTIs are divided into upper respiratory tract infections (throat and sinuses) and lower respiratory tract infections (airways and lungs). The root cause of limitations to progressive airflow is the excessive inflammatory response to breath irritants. The inflammation is due to neutrophils, macrophages, and activated T-lymphocytes (cytotoxic T-cells [Tc1] and helper T-cells [Th1]), leading to emphysema, airway fibrosis, mucus hypersecretion, and oxidative stress. To date, the medical practitioners’ primary focus has been on the antagonists that inhibit the recruitment and activation of inflammatory cells. However, none of the currently available anti-inflammatory medications provide satisfactory relief to COPD patients and may end up producing side effects; therefore, safe, effective medications for inhibiting inflammatory response are needed to treat COPD [4]. An overview of respiratory tract infections caused by bacteria or viruses is depicted in Figure 1.

For thousands of years, herbal drugs have been used to cure numerous illnesses and to improve overall well-being. Among the commonly used herbal medicines, *Panax ginseng* C. A. Meyer is a recognized herb cultivated mainly in Korea, China, and the U.S.A. It is used worldwide to treat many diseases. The principal ingredients of ginseng are amino acids, proteins, flavonoids, volatile oils, and polysaccharides [5,6]. Various forms of ginseng are available, including fresh, dried, boiled, and red ginseng, as well as extracts. These extracts are marketed as dietary, nutritional, and wellness supplements [7,8,9].

In the past 50 years, numerous clinical and preclinical research studies have been conducted on ginseng [10,11]. These have shown that *P. ginseng* improves lung function and overall quality of life compared with pharmacotherapy alone. However, few studies have explored *P. ginseng* against COPD and other associated disorders, such as chronic bronchitis, but these have shown encouraging results [12,13,14,15]. These studies have resulted in a better understanding of ginseng’s phytocomponents, their possible mechanism of action, pharmacological properties, and toxicological profile. The key active component of ginseng was first established by Shibata et al. based on their aglycone moieties as dammarane-type triterpene saponins and ginsenosides. These can be further categorized into 20(S)-protopanaxatriol (ginsenosides Re, Rg1, Rg2, and Rh1) and 20(S)-protopanaxadiol (ginsenosides Rb1, Rb2, Rb3, Rc, and Rd) class [16]. The active constituents’ composition and quality depend on various factors, such as the method of cultivation, harvesting season, preservation method, age, and part of the plant used [17]. Naturally occurring ginsenosides have been shown to possess antifungal and antimicrobial properties. Their bitter taste makes them a suitable antifeedant [17,18,19,20].

Human immune cells were treated with various ginseng extracts by Lau et al. Seven ginsenosides were reported that could specifically suppress production of the inflammatory gene CXCL-10. The observed anti-inflammatory role of ginseng was attributed to the combined effects of these ginsenosides targeting different immunological activity levels, thereby contributing to ginseng’s various actions in humans [21]. Ginseng suppresses a portion of the TNF-α-inducible cytokines and signaling proteins in promonocytic cells and exerts its anti-inflammatory activity by targeting different levels of the TNF-α signaling pathways [22]. Studies conducted on animals have shown that ginseng provokes a robust immune response that protects against bacterial and viral infections [23,24,25]. Ginseng induces the dissolution and dispersion of mature biofilms and also represses biofilm development. The role of ginseng and its main active constituents in reducing the risk and continuation of flu and colds has been reported in several studies, including clinical trials [26]. Various controlled clinical trials have been conducted to study ginseng’s efficacy in COPD, but these trials were small and biased. Therefore, rigorously designed studies are needed [7].

Herein, we reviewed the available literature on ginseng’s active components and their role against respiratory pathogens. The present review summarized ginseng’s possible modes of action, clinical evidence, and consequences as a therapeutic agent against respiratory infections. Interventional clinical trials are needed to evaluate ginseng’s properties, including immunomodulatory, anti-inflammatory, antimicrobial, and antiviral activities.

## 2. The Methodology of the Literature Review

**Figure 2:**
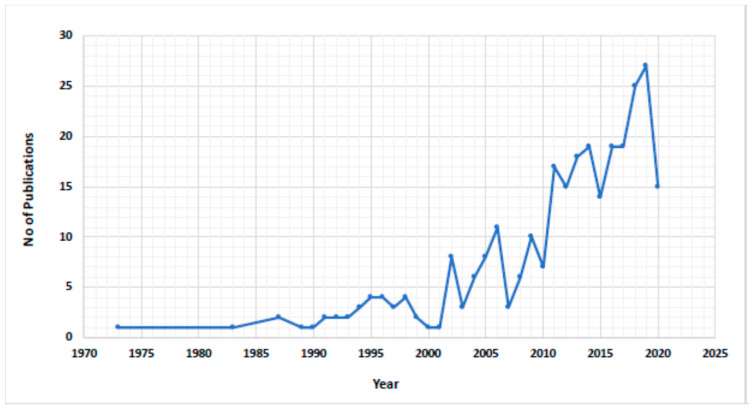
(Search Query: Ginseng in respiratory diseases) The status of publication counts per year Figure 1973.

The literature that was reviewed was found in PubMed, EMBASE, and the Web of Science. The following keywords were used to search the databases: ‘ginseng’, ‘red ginseng’, ‘Korean ginseng’, ‘American ginseng’, and ‘Indian ginseng’. Phrases were also used to search the related literature, such as “ginseng in respiratory disease”, “role of ginseng in COPD”, “ginseng in acute respiratory infections”, and “role of ginseng in respiratory tract infection”. The PubMed database revealed an increasing trend in the number of published articles, particularly since 2000, as shown in Figure 2. Randomized controlled trials (RCTs) examining the role of ginseng vs placeboes in treating respiratory viral and bacterial infections were reviewed in this article. Relevant animal studies were also covered in this review.

## 3. Ginseng Structural Features

**Figure 3:**
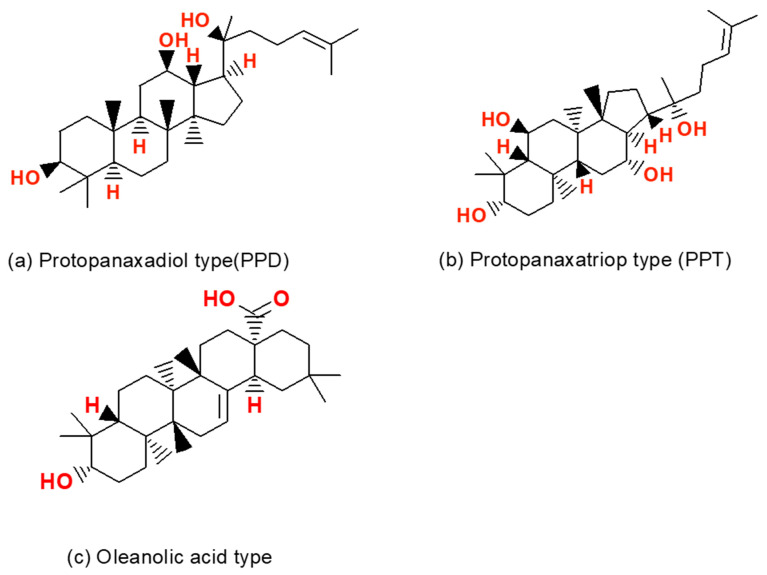
Classification of ginsenosides. Most dammarane-type ginsenosides consist of 17 carbons in a four-ring structure with different sugar moieties. (**a**) protopanaxadiol (PPD), (**b**) protopanaxatriol (PPT), and (**c**) Oleanane types [37].

The primary structural moiety of ginseng saponins is a hydrophobic, four trans-ring rigid steroidal skeleton [27]. Ginsenosides are saponins that are derivatives of triterpene dammarane. Various sugar moieties, such as rhamnose, arabinose, glucose, and xylose, are attached to various carbon positions (C-20, C-6 and C-3) [28]. Each ginsenoside is distinguished by the position of its sugar moiety on the dammarane and triterpene rings. Ginsenosides are generally classified into the following three types: panaxatriol, panaxadiol, and oleanolic [29]. The chemical structures of the three-basic ginseng saponins are shown in Figure 3. The most abundant saponins are protopanaxadiol (PPD; e.g., Ra, Rb, Rc, Rd, Rg3, Rh2) and protopanaxatriol (PPT; e.g., Re, Rf, Rg1, Rg2, Rh1) [30]. More than 40 ginsenosides have been isolated from the root of *P. ginseng* with Rb1, Rb2, Rc, Rd, Rg3, Rh2, Re, Rf, Rg1, Rg2, and Rh1 being the major isolated ginsenosides. Among the various ginsenosides, Rg1, Rg3, Re, and Rd have been the most studied. Most of the studies have focused on the role of ginsenosides, rather than ginseng extract, for treating diseases [6,31,32,33,34,35,36].

## 4. Pathogenicity of Microbial Infections

A family of microbes accountable for diseases is called microbial pathogenicity. A pathogen is a microbe that may produce disease, and the host is an infected organism. A viral or bacterial infection is the leading cause of mortality and morbidity worldwide. This is due to the lack of medicines to combat these infectious diseases. As new contagious diseases emerge, the old ones that were considered to be controlled are re-emerging. Microbes have persisted to adapt, re-adapt, challenge, and survive in the last few decades [38]. Viruses produce respiratory infections by expediting secondary infections, assisting bacterial colonization, adherence, and translocation through the respiratory cells’ epithelial barrier [39]. Air pollution is the most significant cause of respiratory tract infections due to urbanization and industrial development. Clinical features do not distinguish the bacterial and viral infections, but they do have different treatment regimes.

The management and treatment of viral and bacterial infections are complex [2]. Viruses promote superinfection via diverse and replete mechanisms, including infecting airways and disregulating innate and acquired immune responses. They promote bacterial growth, adherence, and incursion into sterile sites within the respiratory tract [40]. Rapid molecular diagnostic tests allow us to identify bacterial and viral pathogens [41]. Correct use of cytological criteria, specimen collection, rapid processing, transport, and antibiotic therapy influence the diagnostic yield [42]. A blood culture is recognized as the gold standard for definitive diagnosis of fungal and bacterial infections worldwide. Bacterial infection, atypical pathogens and bacteria from sterile sites are all confirmed by a blood culture [43]. Molecular diagnostic tests have improved our understanding of the role of viruses in pneumonia, suggesting that we may have underestimated viral pneumonia. A patient’s age, symptoms, radiographic changes, rapid onset of the disease, and a viral epidemic in the community may help differentiate viral and bacterial infections. No consensus has emerged as to whether to treat patients with community-acquired pneumonia with antibiotics [44]. The pathogenesis of bacterial and viral interactions explains respiratory infections and may help in the advancement of new methods of management, prevention, and treatment of acute respiratory infections [26].

## 5. Ginseng’s Immunomodulator Effect

*Panax quinquefolius* L. (American ginseng), *Panax ginseng C. A. Meyer* (Korean ginseng), and *Panax notoginseng* (Chinese ginseng) are the best traditional species of ginseng. They have been shown to possess the potential to improve overall health conditions and mitigate disease symptoms; therefore, these species of ginseng draw special attention [45,46]. *P. ginseng* is plentiful in Korea, China’s eastern region, Russia, and Japan. China mainly cultivates *P. notoginseng* [47,48], while *P. quinquefolium* is cultivated in Canada and the United States, and has been used by Americans for many years [49]. Ginseng contains Gintonin, which is a non-saponin bioactive component. The chemical properties of gintonin are carbohydrates, lipids, and the ginseng protein complex. It is a glycolipoprotein complex that includes three lipid-derived G protein-coupled receptor ligands lysophosphatidylinositols, lysophosphatidic acids and linoleic acid [50].

*Withania somnifera*, commonly called as “Indian ginseng,” is a popular medicinal herb of India. It contains more than 40 withanolides, 12 alkaloids, and uncommon sitoindosides [51,52]. *W. somnifera*’s main active ingredient is withanaloid. Withanaloid (steroidal lactones) is a class of oxygenated ergostane-type steroids that have a lactone in the side chain and a 2-en-1-one system in the ring. These are mainly withaferin A, withanolides A-Y, withasomniferin-A, withasomidienone, withasomniferols A-C, withanone, and etc. The two main withanolides, withaferin A and withanolide D, are responsible for the majority of Ashwaganda’s pharmacological effects. *W. somnifera*’s main alkaloids are anaferine, anahygrine, isopelletierine, pseudotropine, tropine, hygrine, mesoanaferine, choline, withananine, hentriacontane, visamine, withasomnine, cuseohygrine, ashwagandhanolide somniferinine, and somniferiene. Sitoindosides VII, sitoindoside VIII, acylsterylglucosides are the saponins of *W. sominifera* [53,54].

Ginseng exhibits immunomodulatory properties and therapeutic potential against microbial infections. The observed contradiction in ginseng’s immunomodulatory properties stems from the extraction method, differences in the origin, laboratory practices, and source of ginseng [55]. Ginseng is comprised of several pharmacologically active ingredients, such as saponins, phytosterols, acidic polysaccharides, amino acids, nitrogenous substances, polyphenolic compounds, polyacetylenes, vitamins, and minerals. These can treat, alleviate, and offer protection against various diseases [5,37,56].

*P. ginseng* has been used to improve vitality, enhance physical performance, and alleviate stress and aging [57]. Korean ginseng comprises about 200 active compounds, including polysaccharides, amino acids, ginsenosides, and peptides [11]. *P. quinquefolium* is a generally acknowledged herbal remedy and nutritional supplement worldwide [37,58]. Approximately 100 compounds have been isolated from American ginseng. Several differences have been found between American ginseng and Korean ginseng due to their mechanisms of action [59]. However, the primary active components in both are Ginsenosides, Rb1, Rd, Re, Rg1, Rg2, Rg3, Rh1, and Rh2. These components enhance therapeutic activity and stability [60,61]. The pseudoginsenoside F11 in American ginseng and ginsenoside Rf in Korean ginseng and notoginseng differentiate them [59]. Moreover, the ratio of Rg1/Rb1 also differentiates these ginsengs. American ginseng is represented by a ratio of Rg1/Rb1 < 0.4 and Korean ginseng is represented by a ratio of Rg1/Rb1 > 0.4 [61]. Sterols and triterpene glycosides belong to a heterogeneous group of saponins and ginsenoside [62]. After an infection challenge, IL-4 and IL-5 cytokine-producing cells are induced by ginseng. Ginseng polysaccharides demonstrate an immune modulator property [63].

*Panax ginseng* Meyer (*P. ginseng*; *Korean ginseng*) has also been shown to possess medicinal properties. It has been used to treat neurological, cardiovascular, immunological, and autoimmune diseases. It has exhibited neurotherapeutic efficacy in treating multiple sclerosis (MS), amyotrophic lateral sclerosis (ALS), Parkinson’s, Huntington’s, and Alzheimer’s disease [64]. Ginsenosides have shown antioxidant effects in cell cultures and animal models [65,66]. Direct ROS scavenging effects and ligand-receptor signaling have been suggested as possible mechanisms by which ginsenoside (Rb1) exerts its antioxidant activity. Most of the ginsenosides have been identified as phytoestrogens. They possess a four trans-ring rigid steroid skeleton with a modified side-chain at C20, which is missing in 17β-estradiol (E2) [26,27,67]. Ginseng has been shown to possess anti-oxidative and anti-inflammatory properties. It enhances cell-mediated immunity associated with healing and tissue repair, and also reduces the symptoms of respiratory tract infections.

## 6. Impact of Ginseng on Respiratory Virus Infections

**Figure 4:**
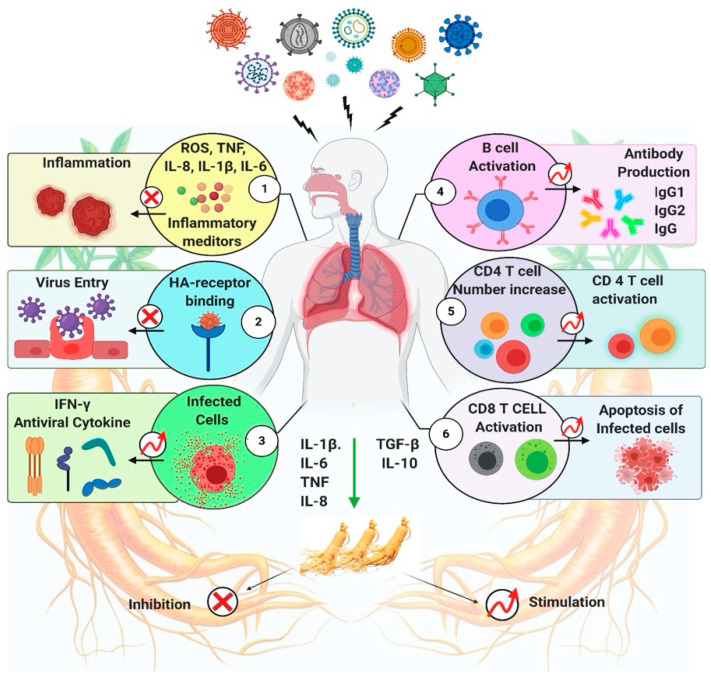
A summary of the impact of ginseng on respiratory virus infections. Ginseng is believed to interrupt through multiple mechanism and shows antiviral effect against respiratory viruses. (**1**) Ginseng shows anti-inflammatory effects by suppressing inflammatory mediators IL-6, IL-1β, IL-8, TNF, and ROS. (**2**) Prevents viral entry into the cells by binding with HA receptor. (**3**) Stimulates the production of IFN-γ. Ginseng induces apoptosis of infected cells by stimulating antibody production (**4**), by activation of CD4+ T cells (**5**), and CD8 + T cells (**6**) [37].

A virus is a small infectious parasite that cannot replicate by itself. Replication of a virus is only possible in a host, such as the living cells of fungi, bacteria, plants, and animals. Viral diseases include immunodeficiency, autoimmune, cancer, organ-specific infectious diseases, diarrhea, influenza, and the common cold. Viruses are characterized by a simple structure and incredibly small size [64,68,69,70,71]. The advancement of vaccines and antiviral therapies has helped us minimize the severity of viral infections and shorten the duration of diseases [72,73,74]. *Ginseng radix* has been used as an herbal medicine to treat respiratory diseases. A specific guideline has been developed for its use in clinical practice. A summary of the impact of ginseng on respiratory virus infections has shown in Figure 4.

### 6.1. Influenza Virus

The influenza virus is a common human respiratory pathogen belonging to the orthomyxoviridae family and is the leading cause of seasonal influenza [75]. This virus is the most common cause of a periodic pandemic and an annual endemic infection. Three types of influenza viruses (A, B, and C) have been found. Human influenza types A and B provoke seasonal infections in winter, while influenza type C induces mild respiratory diseases. Influenza viruses are further classified into subtypes based on the properties of their viral surface proteins hemagglutinin (H) and neuraminidase (N). Lethal strains of the influenza virus have been a cause of deaths worldwide [26,37,75,76,77]. Several studies have established the potential antiviral activity of red ginseng extracts and their purified components against both in vitro and in vivo influenza A. In vitro, mechanistic studies suggest that ginsenosides, particularly Rb1, will interact with viral hemagglutinin proteins to prevent attachment of the H1 N1 virus to α 2–3′ sialic acid receptors on the host cell surface. This results in the minimization of viral entry, thereby decreasing the severity of infection. Fermented Red Ginseng extracts (RGEs), protopanaxadiol [PD], protopanaxatriol [PT], compound K and Rh2 exhibited antiviral activity against various influenza subtypes (H1N1, H3N2, H5N1, H7N9) in mouse models [78].

Intra-nasal inoculation of mice with fermented ginseng extract and the influenza virus showed improved survival rates. It offered protection against H1N1, H3N2, H5N1, and H7N9 influenza strains and showed a dose-dependent efficacy. Fermented ginseng products exhibited superior anti-viral effects against influenza viruses compared with non-fermented ginseng samples [79]. PT-type ginsenoside Re inhibits virus-induced protein production to protect human umbilical vein endothelial cells from avian H9N2/G1-induced cell death. Mucosal adjuvant RGE inhibits influenza virus A/PR8 during viral infections [63]. When inactivated virus and RGE were administered intranasally in mice, researchers observed increased levels of influenza virus-specific antibodies with improved neutralizing activities in blood and mucosal secretions. Specifically, a spike in the IgA antibody was noted in the lungs. This resulted in enhanced secretion of Th1 and Th2-type cytokines in splenocytes in response to the infection challenge. The Th2 type response was more pronounced. The adjuvant effect of RGE was comparable to the effects of conventional adjuvants, such as cholera toxin and aluminum hydroxide.

The role of ginseng in preventing colds has not been clearly determined. Random clinical trials involving ginseng-based proprietary products have provided inconsistent results. A statistically significant reduction in influenza and laboratory-confirmed colds were observed in some of the trials. In contrast, others showed small clinical changes only in upper respiratory tract infections that were not laboratory-confirmed [80].

A double-blind, placebo-controlled four-week study of 227 participants was conducted. Half of the participants were given ginseng at a dosage of 100 mg daily and the other half a placebo. All participants received the influenza vaccine. The finding showed a significant lessening in the frequency of colds and flu in the treated group over the placebo group, and antibody determinations in response to the vaccination were higher in the treated group than in the placebo group [81]. Similarly, North American ginseng has been suggested to help prevent and treat viral respiratory illnesses [82]. The stems and leaves of *Panax ginseng* showed an adjuvant effect on poultry vaccines [83]. Ginseng stem-leaf saponins (GSLS) combined with selenium have exhibited an adjuvant effect on the live vaccine of Newcastle disease virus, infectious bronchitis virus, and intraocular and intranasal immunization in chickens [84].

### 6.2. Respiratory Syncytial Virus

Respiratory syncytial virus (RSV) belongs to the *Paramyxoviridae* family. This virus has been shown to cause seasonal pandemics, acute bronchiolitis, and newborn viral deaths [37]. Airborne droplets and close contact can transmit this virus. The symptoms of this viral infection include laryngitis, pharyngitis, rhinitis, and high fever, followed by pneumonia, bronchitis, and often death. Newborns show heavier symptoms, while upper respiratory tract infection is the primary manifestation of the infection in older children and adults [85]. The respiratory syncytial virus mainly affects patients with congenital heart diseases and immunocompromised individuals. To date, no vaccine is available against RSV, despite extensive research efforts [86].

RSV is a negative-sense single-stranded RNA virus. Eleven proteins are coded by its genome, of which the surface proteins G and F are essential for RSV binding and fusion, respectively. Of the two proteins, the former is antigenically variable, while the latter is highly conserved. The F protein has a conformational structure which is different before and after fusion. In addition to this, it performs an unpredictable switching between the two structures. The symptoms of bronchiolitis usually peak around four to six days of illness; the peak symptoms cause the airway obstruction due to the host’s inflammatory immune response. At present, no curative therapies are available, and the guidelines only cover supportive management of RSV [87]. Viral, host, and environmental factors contribute to disease severity and RSV pathogenesis [88]. Infants infected by RSV bronchiolitis were shown to have a higher level of interleukin 8 (IL-8) than the control group [89]. RSV infection caused an increase in the levels of cytokines (IL-6, IL-8, IL-10), chemokines, and tumor necrosis factor-alpha (TNF-α) in different vitro models and resulted in an imbalance in type1 and type 2 cytokines [89,90,91,92].

In many infectious diseases, ginsenosides and ginseng increase Th cells’ immune response by improving the cytokines production of Th1 (IL-2, TNF-α, IFN-γ,) and Th2 (IL-4, IL-10, and IL-13). Ginseng inhibits bacterial infection in mice by increasing serum antibodies IgG1 and IgG2a [93]. The efficacy of ginseng in human clinical trials has been proved via in vivo and in vitro experiments, demonstrating an improved immune response against infections [37]. Ginseng-induced nitric oxide production relaxes the airways and also contracts smooth muscles. Anti-inflammatory and antitumor effects have been exhibited by the Ginsenoside Rh2 (G-Rh2), an active ingredient of ginseng. G-Rh2 has been shown to control allergic airway inflammation by regulating NF-κB activation and p38 MAPK phosphorylation [94]. Ginsenoside Rg3 suppresses the matrix metalloproteinase (MMP-9) activity and inhibits oxidative stress (superoxide, NO and iNOS) [95]. Ginsenoside RG-II increased pro-inflammatory interferon-gamma (IFN-γ) levels, but lowered the production of IL-4 [96].

### 6.3. Rhinoviruses

Human rhinoviruses (RV) are positive-stranded RNA viruses which cause respiratory tract diseases in children and adults. RV is the causative agent for the common cold in healthy individuals and triggers only mild upper respiratory tract infections. This virus can also cause long-lasting and severe pulmonary infections in people who are immunocompromised and suffering from chronic lung diseases. [26,97]. Acute exacerbations of asthma are also caused primarily by rhinoviruses (RVs). An increase in submucosal CD3+ lymphocytes and eosinophils were reported in two studies about rhinovirus-induced airway inflammation in normal and asthmatic subjects. The increase in the number of mucosal CD3+ cells was associated with a corresponding increase in airway responsiveness and correlated positively with cold symptoms [98]. To date, no remedy has been found to treat rhinovirus infection.

Protopanaxatriol (PT)-type ginsenosides (Re, Rf, and Rg2), and protopanaxadiol (PD)-type ginsenosides (Rb1, Rb2, Rc, and Rd) have been studied for their antiviral activity against rhinovirus infection. PT-type ginsenosides were found to protect HeLa cells from human rhinovirus 3 (HRV3)-induced cell death, as determined by sulforhodamine B staining of viable cells and morphological assessment. In contrast, PD-type ginsenosides did not exhibit protective effects and significantly promoted HRV3-induced cell death, indicating a structure-dependent effect for ginsenosides on HRV3. In the case of coxsackievirus, the panaxatriol-type ginsenosides demonstrated limited antiviral behaviours [99].

Korean red ginseng (KRG) showed antiviral activities in a study examining its effects on primary human nasal epithelial cells in human rhinovirus. KRG was used to treat subsequent HRV (human rhinovirus) infection in a study that measured the mRNA and protein levels of the inflammatory cytokine’s interleukin IL-8 and IL-6 using real-time polymerase chain reaction and enzyme-linked immunosorbent assay. In primary HNE cells, KRG significantly lessened the HRV-induced upregulation of IL-8, IL-6 mRNA and protein levels. Rhinovirus-induced NF-κB and MAP kinase activation was also inhibited by KRG treatment. KRG might reduce inflammatory responses to HRV infection, as well as prevent HRV-induced asthma exacerbations [100,101]. Ginsengs were found to affect membrane penetration, inhibit replication inside the cells, and hinder viral attachment.

### 6.4. Coronaviruses

On 11 March 2020, the WHO declared COVID-19 a pandemic. Five pandemics have been globally circulated by a virus since 1918. COVID-19 emerged as the third coronavirus in the past two decades [102,103,104]. The previous two acute respiratory syndromes, (SARS)-CoV and the Middle East respiratory syndrome (MERS)-CoV, were responsible for severe health issues. Coronavirus spread worldwide from human to human through droplets of infected patients’ sneezing and coughing [105]. The common symptoms of COVID-19 are a cough, chest tightness, dyspnea, and fatigue, further manifesting into septic shock or sepsis which could cause death [106]. *Panax ginseng* may be useful here as an immunomodulator and for preventive and supportive therapy [107].

As a treatment for upper respiratory tract diseases, ginseng has been used for thousands of years. Ginseng is capable of binding to the angiotensin-converting enzyme 2 (ACE2) receptor. ACE2 is not the only entry receptor of the coronavirus. COVID-19 might be treated by inhibiting excessive immune cell activation and cytokine production, so ginseng may enhance the immune response, as it has cytokine-modulation activity. Herbal medicines which inhibit 1L-1, IL-6, TNF-α, and other pyrogenic cytokines by inhibiting the cytokine storm may also be effective against COVID-19. Several ginsenosides have been recognized as phytoestrogens, binding estrogen receptors [108,109,110]. These risk factors contribute to a higher concentration of reactive oxygen species and oxidants [111,112,113]. Antioxidant enzymes stimulate estrogen, which acts as an antioxidant, lessening ROS production [27,110]. American ginseng has shown antimicrobial effects against numerous strains of bacteria and also alleviates the detoxifying enzymes, along with the level of reactive oxygen species [114].

Numerous studies have shown the effectiveness of American ginseng extract in treating influenza-infected older adults. It has reduced the incidence of acute respiratory infection in vaccinated and non-vaccinated individuals [114,115,116,117]. Ginsenoside Rb1 efficiently lessens ROS production-induced risk factors such as TNF-α [114,118,119]. Researchers have suggested that viral infection and immune dysfunctions are due to chronic fatigue syndrome (CFS). It is anticipated that ginsenoside Rg1 reduces the peroxidation product malondialdehyde production and increases nerve cells’ antioxidant capacity. It also reduces free radical production in chronic fatigue syndrome, an actual cause of viral infections [120,121]. A study was conducted with 100 volunteers to check the efficacy of Korean Red Ginseng’s (KRG) against acute respiratory illness. The results suggested that KRG may provide protection from acute respiratory illness or reduce its symptoms [122].

**Figure 5:**
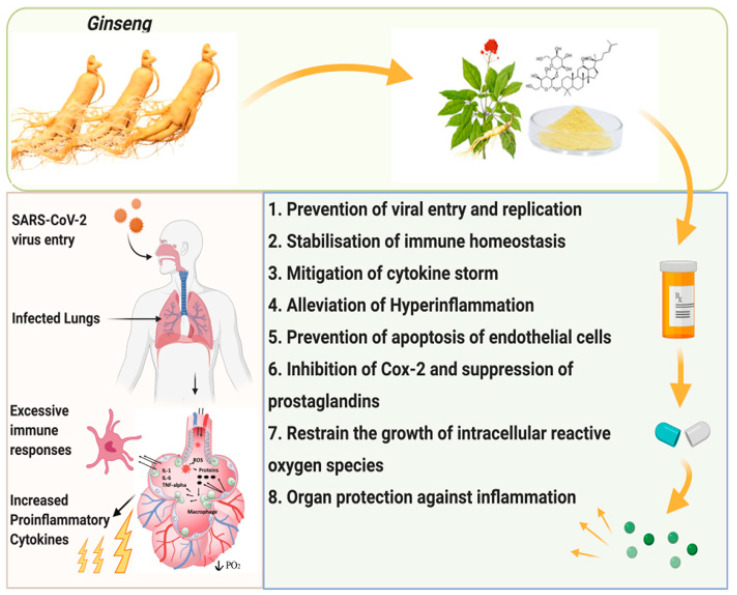
Probable role of Ginseng for coronavirus.

In clinical trials and animal experiments, ginseng has been found to improve protection against pneumococcal pneumonia and influenza. Hence, based on these findings, ginseng may help provide immunity against COVID-19 [123]. *W. somnifera* is utilized to treat nearly all illnesses that affect human health because of its broad area of activity. Several studies have shown that derivatives of *W. somnifera* can efficiently inhibit various viral infections, including HPV, herpes simplex, parainfuenza-3, HCV, H1N1, bursal disease viruses, and coronaviruses including SARS-CoV and SARS-CoV-2 [124,125,126,127,128,129]. Since *W. somnifera* has antiviral, immunomodulatory, anti-inflammatory, and prophylactic properties, the Indian government (Ministry of Ayurveda, Yoga & Naturopathy, Unani, Siddha, and Homoeopathy), as well as the Council of Scientific and Industrial Research and the Indian Council of Medical Research, have recently approved its use in clinical trials against SARS-CoV-2 [130]. Table 1 shows the effects of ginseng on different respiratory virus infections. The probable role of ginseng for coronavirus is shown in Figure 5 [131]. A fourteen-day clinical trial was recently conducted to study the efficacy of the Shenhuang granule containing *Panax ginseng*, *Rheum palmatum* L. stem, *Sargentodoxa cuneata*, *Taraxacum mongolicum*, *Aconiti lateralis*, *Radix praeparata*, and *Whitmania pigra*. The trial showed improvement in the condition of severe COVID-patients [106]. Thus, it is advisable to choose a reliable ginseng product as adjuvant therapy for COVID patients.

## 7. Anti-Bacterial Activity of Ginseng

Microbial infections have various causes, and the resulting diseases require different antibiotics as treatment. However, the improper use of antibiotics is the cause of resistance and toxic side effects, as well as the emergence of multidrug-resistant bacteria, which is now a global health emergency [144]. In the absence of newer antibiotics, natural products are being promoted to address this issue. Ginseng has been reported to inhibit bacterial pathways, thereby killing bacteria indirectly. It has also been shown to protect the host from bacterial invasion [145,146].

**Figure 6:**
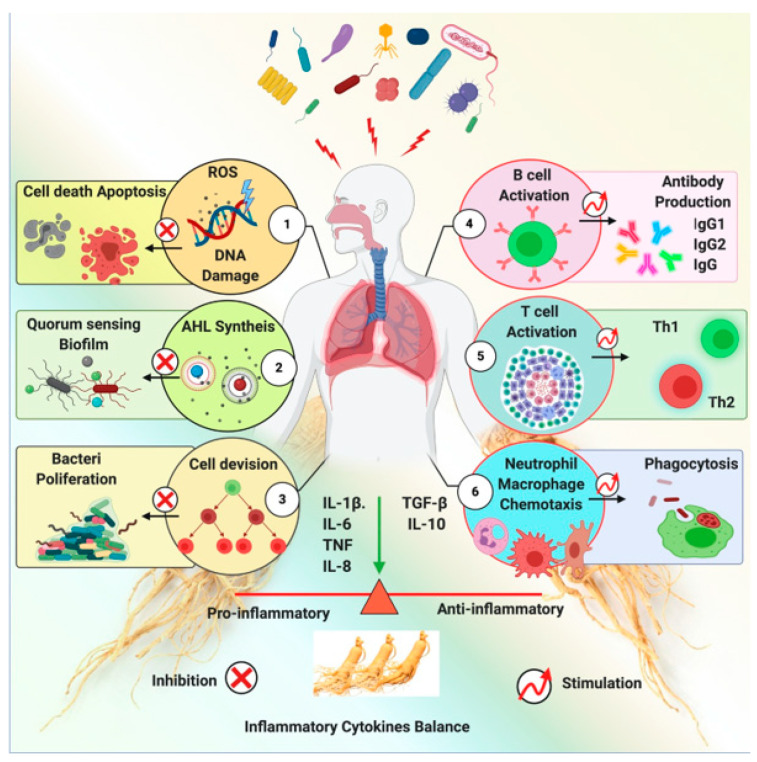
Ginseng and its derived components’ anti-bacterial effects via multiple mechanisms. (**1**) Ginseng inhibits the DNA damage and apoptosis by inhibition of ROS, (**2**) suppresses AHL-(acyl homoserine lactones) leading to the inhibition of quorum sensing biofilm formation of bacteria, (**3**) inhibits cell division and bacterial proliferation, (**4**) stimulates B cell activation and antibody production, (**5**) activates Th1 and Th2 response, (**6**) ginseng enhances phagocytosis of Neutrophil and macrophage [37].

Ginseng exhibits a shielding effect against the inflammation induced by a pathogen. Ginseng exerts this effect via several mechanisms, including anti-quorum sensing, inhibition of pathogen-induced hemagglutination, DNA mutagenesis, and immune-modulatory functions. An impression of ginseng’s antibacterial activity is shown in Figure 6. Ginseng and its derived components’ anti-bacterial effects are represented in Table 2.

### 7.1. Pseudomonas Aeruginosa

Pseudomonas is commonly found in soil, water, and the environment. When people come in contact with this contaminated water or soil, they become infected [37,163]. While multiple types of *Pseudomonas* exist, *Pseudomonas aeruginosa* causes most of the infections in humans. This type causes infection in the lungs (pneumonia), but it has evolved to circumvent the effects of the antibiotics used to treat it [26,37,146].

Kharazmi et al. studied the effect of *P. ginseng* in the treatment of *Pseudomonas aeruginosa* infection in rats. *P. ginseng* aqueous extract was administered by subcutaneous injection at a dose of 25 mg/kg of body weight per day, along with saline as a control. After two weeks of treatment, the study showed a significant increase in blood polymorphonuclear leukocyte (PMN) chemiluminescence (*p* ≤ 0.001) with a decrease in the level of serum immunoglobulin G (IgG) against *P. aeruginosa* (*p* < 0.05). The ginseng-treated infected group showed a higher IgG2a level and lower IgG1 level than the control group. In the ginseng-treated group, the macroscopic lung pathology was milder, with a lower percent of PMNs in the cells collected by broncho-alveolar lavage. The variations in IgG1 and IgG2a subclasses imply a possible shift from a Th-2- to a Th-1 response. The findings of this study suggested that the effect of *P. ginseng* could be related to the activation of a Th-1 type of cellular immunity and down-regulation of humoral immunity [164]. *P. ginseng* might also be considered an add-on therapy to treat cystic fibrosis, as it can reduce bacterial infections and biofilm formation.

Another study was conducted to investigate the antimicrobial activity of the aqueous extract of *Panax quinquefolius* from North American ginseng (NAGE) root against *Pseudomonas aeruginosa.* MIC (minimum inhibitory concentrations) of reference and *Pseudomonas aeruginosa*’s clinical isolates were measured by a standard agar dilution method. The extract eradicated six-day-old mature biofilms (5% *w*/*v*), while luorescence microscopy displayed a reduction of live cells and biofilm complexes compared with non-treated biofilms [165].

Ginseng is a complex mixture of several components, some of which enhance bacterial growth, while others repress it. Thus, the separate components need to be studied. Previous studies via animal models showed that ginseng treatment offered protection from chronic lung infection caused by *P. aeruginosa*. Wu et al. studied ginseng’s effect on *P. aeruginosa* motility and biofilm formation (in vitro and in vivo). Ginseng extract was found to enhance the swimming and twitching motility but reduced the swarming motility. However, an aqueous extract of ginseng in concentrations of 0.5–2.0% did not inhibit *P. aeruginosa*, but it did significantly limit the formation of *P. aeruginosa*’s biofilm. This study’s results suggested that ginseng might alleviate biofilm-associated chronic infections caused by *P. aeruginosa* [155]. Ginseng was reported to have a negative effect on the quorum sensing system of *P. aeruginosa.* This was suggested as a possible mechanism noted in a previous study by which ginseng helped the bacterial clearance from animal lungs in vivo. The ability to enhance and repress bacterial growth could be mutually exclusive [166].

HPLC analysis of ginseng’s hot water extract has revealed the presence of six ginsenosides. Therefore, it is essential to isolate and evaluate ginseng’s components which contribute to its anti-quorum sensing activity. Ginseng’s immunomodulatory functions most probably associate with the activation of Th1 type cellular immune response. These functions deregulate the humoral immune response and lessen the formation of immune complexes [126,137]. Ginseng could play a vital role in combating microbial infections, particularly against *P. aeruginosa* pneumonia. PMNs are a common cause of cystic fibrosis, the leading cause of morbidity and mortality [167,168]. Thus, ginseng shows good therapeutic activity against *P. aeruginosa* pneumonia [169].

### 7.2. Streptococcus Pneumonia

*Streptococcus pneumoniae* (*S. pneumoniae*) are gram-positive, lancet-shaped, facultative-anaerobic bacteria with over 90 known serotypes. Most of the *S. pneumoniae* produce diseases; a few of the serotypes cause most of the pneumococcal infections. *Streptococcus pneumoniae* have caused diseases all over the world [146]. The human respiratory tract has commensal Pneumococcus, which is the cause of local infections, as well as many invasive diseases, such as meningitis and sepsis, due to its virulence factors. Korean Red Ginseng (KRG) has been reported to enhance the efficacy of the pneumococcal *pep27* mutant (Δpep27) vaccine [170]. KRG, at a dose of 100 mg/kg, demonstrated higher survival rates in mice than the non-treated controls. Additionally, pre-treated mice showed lower morbidity and bacterial numbers. In vivo, the dosing of 100 mg/kg of KRG lowered cytokine levels, TNF-α and IL-1β, nitric oxide level, and post-infection 48 h neutrophil infiltration. KRG was shown to defend the host cells from lethal pneumococcal sepsis and also enhanced bacterial clearance. It thereby strengthens cell continuance against pneumococcal infection [171].

Korean Red Ginseng also significantly reduced NF-κB, TLR2, and TLR4 appearance in RAW 264.7 macrophages induced by *S. pneumoniae.* Korean red ginseng extract’s protective effect against pneumococcal infection and sepsis have been investigated. In one study, mice were administered KRG (25, 50, 100 mg/kg) for 15 days, then infected with a lethal *S. pneumoniae* strain. Colonization, survival rate and body weight were calculated. Mice treated with 100 mg/kg of KRG had significantly higher survival rates and body weights than those of the non-treated controls. A dosage of 100 mg/kg of KRG protected the host cells from fatal pneumococcal sepsis by inhibiting inflammation and intensifying bacterial clearance, augmenting cell survival against the pneumococcal infection [172].

## 8. Ginseng Clinical Trials

In this section, summaries of human clinical trials from various databases, such as lens.org and clinicaltrial.org, are presented. Only fifteen studies were found for ginseng and respiratory diseases, as noted in Table 3. Ginseng’s clinical trials were heterogeneous concerning their variety, species, duration, dose, indications, and participant characteristics. However, in order to illustrate the efficacy and safety of ginseng, future studies need to be stricter and methodologically relevant. No formal inventory has been created showing ginseng in the context of respiratory diseases. Ginseng products are generally used as complementary and alternative medicine in respiratory infections. Most clinical trials have been conducted on *P. ginseng* and included a relatively small number of subjects, ranging from healthy adults to patients with symptoms. More research is needed to explore the uses of ginseng in the context of respiratory diseases.

### Evaluations of Ginseng’s Impact on Respiratory Pathogens in Human Clinical Trials

Ginseng has been used for centuries to cure various diseases. Ginseng’s effectiveness has been evaluated in numerous clinical trials exploring treatment of the common cold and flu. Results have shown that ginseng relieves the symptoms and prevents respiratory infections. American ginseng’s constituent COLD-fX (CVT-E002) is known as poly-furanosyl-pyranosyl-saccharide. COLD-fX has been isolated from the roots of American ginseng. It is effective and safe against respiratory pathogens, as well as in reducing the viral load of patients who are prone to seasonal influenza. The immunomodulatory constituents of COLD-fX act through toll receptors and influence a rise in cell numbers and functions in innate and adaptive immune systems [180].

A randomized, double-blinded trial investigated the effectiveness of COLD-fX in acute respiratory illness (ARI). Forty-three adults were given a 200 milligram/capsule of COLD-fX or a placebo twice every morning for four months. After one month, the subjects were administered a standard dose of influenza vaccine. The authors did not evaluate the mechanism by which COLD-fX reduced the incidence and duration of ARI in this study. According to the authors, the immune-modulating properties of COLD-fX reported in earlier studies were probably responsible for the observed effect. After the dosing of COLD-fX in mice in vitro, COLD-fX (CVT-E002) was reported to cause a significant increase in lymphocyte proliferation and cytokine production (IL-1, IL-6, TNF-α, and nitric oxide) from peritoneal macrophages. The extract’s ability to stimulate IL-2 and IFN-γ release could be attributed to its efficacy against respiratory infections. T cell (IFN-γ, IL-2) and NK cytokines correlate with virus-elicited adaptive immunity. Additionally, COLD-fX reduced the relative risk and duration of respiratory symptoms by 48% and 55%, respectively, in immunocompromised, aged patients during the influenza season [117]. COLD-fX was also investigated as a seasonal protective drug on 783 patients aged sixty-five years and above. A dosage schedule of 400 or 800 mg/day was given for six months. It was found that COLD-fX was safe and reduced the severity and incidence of upper respiratory tract infections [174].

Seida et al. studied ginseng’s efficacy in preventing common colds in healthy adults. A systematic review of randomized controlled trials or controlled clinical trials comparing Asian ginseng (*Panax ginseng*) and North American (*Panax quinquefolius*) ginseng root extract to placebo or no treatment groups of healthy adults was carried out. Five trials involving 747 participants were selected for the review. These five trials investigated only North American ginseng and the trials differed in their methodological quality. However, in comparison with the placebo groups, ginseng medications reduced common cold symptoms by 25%. The study noted a trend towards a lower incidence of at least one common cold or other respiratory infection in the ginseng-treated group compared with the placebo group. Ginseng lessened the duration of ARIs or colds by 6.2 days as compared with the placebo [175].

Predy et al. studied the efficacy of North American ginseng containing poly-furanosyl-saccharides in preventing upper respiratory tract infections. At the beginning of influenza season, a double-blind, randomized, placebo-controlled study was conducted. The trial participants, who had a history of at least two colds in the previous year, were chosen from Edmonton, Alberta. The total number of participants was 323, aged from 18 to 65 years. Participants were given two capsules of North American ginseng extract or placebo daily for four months. A moderate dose of North American ginseng for four months lessened the number of colds per person. The results also showed that participants who had two or more prior cold symptoms had less-severe symptoms than those with no prior symptoms [116].

Mono-preparations of ginseng behave as a placebo, as reported in several clinical trials. The adverse events typically reported by the trials are gastrointestinal disorders, sleepiness, and headache. Isolated cases reported more serious adverse events, but it is difficult to provide evidence of casualty. Ginseng as an add-on therapy has shown severe adverse events and even casualties; however, after reviewing all the cases, it is difficult to conclude that *P. ginseng* could cause the problems. Combination therapy does appear to be more closely associated with adverse events [15,181].

A pilot study of a randomized, controlled trial was conducted to evaluate the efficacy of GINST and GS-3K8 modified ginseng extracts in acute respiratory illness. The study showed that both extracts had a positive impact on ARI treatment, demonstrating reduced symptoms. A more randomized controlled trial is needed to validate these results [180]. A *P. ginseng* (G115) dose of 100 mg twice daily for 12 weeks improved the pulmonary function test of respiratory endurance in 92 patients with COPD [166]. In two groups of patients [(n = 37) (n = 38)], the first group was given 875 mg amoxicillin and 125 mg clavulanic acid, while the second group was given an anti-bacterial treatment with 100 mg standardized ginseng extract G115 twice daily for nine days. The bacterial clearance was significantly faster for the ginseng group as compared with the antibiotic-only treatment group. G115 ginseng extract reduced the bacterial counts in patients’ bronchial systems. Those patients who have complicated bacterial clearance may receive benefit from ginseng [14]. Additionally, no side effects were reported in the treatment group receiving *P. ginseng* [15].

Xue et al. evaluated the curative power and safety profile of standard root extract of ginseng (*Panax ginseng* C.A Meyer). They studied the role of ginseng extract in improving the quality of life and providing symptomatic relief. The research design was a multi-centered, randomized, double-blind, placebo-controlled, two-armed parallel clinical trial. The trial involved a total of 168 participants and was conducted at two trial sites in Melbourne, Australia. The participants received 100-mg ginseng capsules or matching placebo twice daily for 24 weeks. The initial result was based on three validated questionnaires of quality of life, and secondary outcomes were based on relief medication usage, lung function testing, exacerbation frequency, and safety. The results of this trial indicated that for chronic respiratory diseases, new remedial development was a possibility. The trial also suggested that ginseng treatment was safe and had remedial value, as it provided symptomatic relief in patients with COPD [7]. In summary, the results of all clinical trials have shown that ginseng and its compounds have protective effects against infectious diseases. As a remedial treatment in respiratory infections, ginseng shows potential for the development of new herbal medicines. More effective clinical trials are still needed to prove the potency and effectiveness of ginseng against respiratory infections.

## 9. Conclusions

Ginseng has been used for centuries as a traditional medicine to cure numerous diseases, but few clinical research studies have been done on its effect on respiratory infections. Most clinical trials have focused on studying the probable effects of ginseng on fatigue, memory, menopause symptoms, and mild diabetes symptoms. Between 2002 and 2017, one hundred and thirty-four trials were registered for ginseng, of which 60.4% were completed and the remaining 23.1% are actively recruiting participants. Only 15 were registered for lung diseases, demonstrating that ginseng’s validity as a remedy for respiratory infections is less well known. However, Panax species phytochemicals are being studied for their natural properties to cure respiratory infections. The stem and leaves have more phytochemicals than the roots, so these plant parts also need to be explored.

Many observed effects have been attributed to *P. ginseng* and its constituent, ginsenosides. It seems that ginsenosides inhibit DNA binding, kinase phosphorylation, ERK1/2, NF-κB transcription factor, and MAPK induction/translocation as these are functional ligands of glucocorticoid receptors. Ginsenosides reduce proteases such as MMP-9 and pro-inflammatory mediators (ROS, IL-6, IL-8, TNF-α). *Panax ginseng* lessens the production of oxidants and enhancing anti-oxidative enzymes to protect against oxidative stress. It can be concluded that respiratory pathogenesis appears to be inhibited by *Panax ginseng* and ginsenosides; therefore, they could be a promising remedy for the treatment of respiratory diseases.

Herbal medicines and natural plant products are less understood mechanistically, so close attention is needed to understand ginseng’s side effects. However, most of the articles suggest that ginseng does not cause adverse effects; it is very well tolerated and efficient in preventing seasonal respiratory infections for all age groups. At the same time, it is necessary to analyze and understand its molecular mechanisms, concentrating on the proteomics procedures, pathway analysis, and applicability of molecular biology’s techniques for the identification of the main cellular markers which trigger anti-oxidative and anti-inflammatory effects. It could be helpful to fix those determinants which are accountable for adverse events by frequent use and continued ingestion of standardized ginseng extracts.

Despite the use of ginseng products as alternative and complementary medicine worldwide, a limited number of clinical trials have investigated the efficacy of *P. ginseng*. In these clinical trials, the number of participants was limited but did include healthy subjects and patients with symptoms. Numerous distinct ginsenosides are present in ginseng extracts; thus, more evaluation is required to reveal their full anti-infective potential. More rigorous and methodological research is required for the evaluation of ginseng’s effectiveness in respiratory infections.

## Figures and Tables

**Table 1:** Effects of ginseng on different respiratory viruses’ infections.

Ginseng Extracts and Compounds	Respiratory Viruses	Study Type	Observations	Conclusions	Reference
seven compounds- mainly belonging to *P. ginseng*	Coronavirus	Glide docking program was utilized for molecular docking	Floralginsenoside B, which is extracted from *Panax ginseng*, indicated a docking score of −8.61 Kcal/mole and showed high binding affinity by interacting with active pocket residues of 6M0J mainly through hydrogen bonds with Gln474, Cys 480, Gly 482, Glu465, and Asp467 than other compounds against the SARS-CoV-2 Spike RBD.	The extracts and essential oils of *Panax ginseng* could be introduced as promising COVID-19 inhibitors.	[132]
*Withania somnifera* (Indian ginseng)	Coronavirus	Molecular docking and dynamics studies	Two different protein targets of SARS-CoV-2, namely NSP15 endoribonuclease and receptor binding domain of prefusion spike protein from SARS-CoV-2, were targeted. Molecular docking studies suggested Withanoside X and Quercetin glucoside from *W. somnifera* have favorable interactions at the binding site of selected proteins, that is, 6W01 and 6M0J.	Based on proven therapeutic potential against n-CoV-2 proteins, Indian ginseng could be one of the alternatives as an antiviral agent in the treatment of COVID-19.	[133]
*Panax ginseng* and *Schizonepeta tenuifolia*	SARS-CoV-2 and Infuenza A viruses.	In-vitro, Cells and cell culture, plasmid transfection and virus assembly, Cytotoxicity assays, Virus infection and drug inhibition assays	RDS contains broad-spectrum antiviral activity, blocking the infection of SARS-CoV, SARS-CoV-2, and Infuenza A viruses.	RDS may broadly inhibit the infection of respiratory viruses such as SARS-CoV, SARS-CoV-2, and Infuenza A.	[134]
Ginseng stem-leaf saponins (GSLS) in combination with selenium	Newcastle disease virus and infectious bronchitis virus	Female yellow chickens	In-vitro, Hemagglutination inhibition test, Immunohistochemical staining for IgG+, IgA+ and IgM+ cells, sIgA assay, RT-qPCR, Transcriptome analysis.	Enhanced antibody responses in GSLS-Se group may be attributed to the immunomodulatory effects of GSLS-Se on the immune-related gene profile expressed in the immunocompetent cells of the HGs.	[84]
Ginseng stem-and-leaf saponin (GSLS)	Newcastle disease virus	White layer chickens	Experiment design, Hemagglutination inhibition test, Immunohistochemical staining for IgG+, IgA+ and IgM+ cells, sIgA assay, RT-qPCR, Transcriptome analysis.	GSLS could be a useful oral adjuvant to improve vaccine immunization in chickens.	[135]
Extract of Korean red ginseng (RG)	Influenza A virus	In vitro and In vivo mice model	Polysaccharide fraction was most effective in reducing the accumulation of (TNF-α)/(iNOS)-producing dendritic cells (tip DCs) in the mouse lungs.	Polysaccharides of RG have a pronounced beneficial effect on the symptoms of influenza virus infection.	[136]
Extract of Korean red ginseng	H1N1 and H3N2 influenza viruses	In vitro, Naive mice model	Red ginseng extract showed significantly enhanced protection, lower levels of lung viral titers and interleukin-6, but higher levels of interferon-γ compared with control mice having virus infections without red ginseng extract.	Intake of ginseng extract will have beneficial effects on preventing lethal infection with newly emerging influenza viruses.	[137]
*Panax ginseng* polysaccharide (PGP)	H1N1 (A/PR/8/34) and H3N2 (A/Philippines/82) influenza viruses	In vitro, mice study	PGP solution showed moderately enhanced survival rates and lower levels of lung viral titers and the inflammatory cytokine (IL-6).	This study demonstrated that PGP can be used as a remedy against influenza viral infection.	[138]
Black ginseng (BG) and red ginseng (RG)	A(H1N1) pdm09 (A/California/04/2009) virus.	In vitro, mice study	BG displayed a 100% survival rate against infection, while mice treated with RG had a 50% survival rate.	BG may be useful as an alternative antiviral adjuvant to modulate immune responses to influenza A virus.	[139]
Fermented ginseng extracts	Different strains of influenza viruses, H1N1, H3N2, H5N1, and H7N9.	Different genetic backgrounds of mice and in the deficient conditions of key adaptive immune components (CD4, CD8, B cell, MHCII)	In vitro cell culture experiments showed moderate virus-neutralizing activity by fermented ginseng extract, probably by inhibiting hemagglutination and neuraminidase activity.	Fermented ginseng extracts might provide a means to treat influenza disease regardless of virus strains.	[79]
Red ginseng extract (RGE)	influenza A virus	In vivo and in vitro, mice model	RGE was found to improve survival of human lung epithelial cells upon influenza virus infection. Also, RGE treatment reduced the expression of pro-inflammatory genes (IL-6, IL-8).	RGE might have the potential beneficial effects on preventing influenza A virus infections via its multiple immunomodulatory functions.	[90]
Ginseng extract and ginsenosides	Influenza A virus	In vivo and in vitro, mice model	Ginsenosides protected the animals from lethal 2009 pandemic H1N1 infection and lowered viral titers in animal lungs.	Ginsenosides are promising candidates for the development of antiviral drugs for influenza viruses.	[78]
Ginseng	Respiratory syncytial virus	BALB/c mice after RSV infection	Ginseng-treated mice that were non-immunized or previously immunized with FI-RSV showed improved protection against RSV challenge compared with control mice without ginseng treatment.	Ginseng can modulate host immune responses to FI-RSV immunization and RSV infection, resulting in protective effects against pulmonary inflammatory disease.	[140]
Panax Korean red ginseng extract (KRGE)	Respiratory syncytial virus	In vitro and in vivo	KRGE improved the survival of human lung epithelial cells against RSV infection and inhibited RSV replication.	Results suggested that KRGE has antiviral activity against RSV infection	[141]
Red ginseng extract (RGE)	RSV	In vitro cell culture and in vivo mouse models	RGE treatment improved lung viral clearance and enhanced the production IFN-γ in bronchoalveolar lavage cells upon RSV infection of mice.	Ginseng has protective effects against RSV infection through multiple mechanisms.	[23]
Seven ginsenosides	Human rhinovirus	Assays for antiviral activity and cytotoxicity were carried out by the sulforhodamine B method using the cytopathic effect (CPE) reduction assay.	The antiviral assays demonstrated that, of the seven ginsenosides, the PT-type ginsenosides (Re, Rf, and Rg2) possess significant antiviral activities against CVB3 and HRV3 at a concentration of 100 μg/mL. Only ginsenoside Rg2 showed significant anti-EV71 activity with no cytotoxicity to cells at 100 μg/mL	Ginsenosides Re, Rf, and Rg2 have the potential to be effective in the treatment of CVB3, EV71, and HRV3 infection.	[142]
*Withania somnifera* (Indian ginseng)	H1N1 Influenza virus	In silico study	High binding affinity of the WA toward NA and revealed several interesting molecular interactions with the residues which are catalytically important during molecular dynamic simulations.	Several interesting molecular interactions with the residues which are catalytically important during molecular dynamic simulations.	[143]

Abbreviations: HG: Harderian gland; RDS: Respiratory Detox Shot; GSLS: ginseng stem-and-leaf saponins; TNF-α: Tumor Necrosis Factor-alpha; iNOS: inducible nitric oxide synthase; PGP: *Panax ginseng* polysaccharide; FI-RSV: Formalin-inactivated respiratory syncytial virus; IFN-γ: Interferon-Gamma; BALB: Bagg Albino (inbred research mouse strain): IgA + cells, Immunoglobulin A-secreting cells; sIgA: Secretory Immunoglobulin A; NA: Neuraminidase; AI: Avian Influenza; CPE; cell viability and cytopathogenic effect; HRV3: Human rhinovirus 3.

**Table 2:** Effects of ginseng on bacterial infections of the respiratory tract.

Ginseng Extracts and Compounds	Microbe	Study Type	Observations	Conclusions	Reference
Withaferin A (WA), a withanolide purified from *Withania somnifera*	*H. pylori*	In vitro study	WA inhibits *H. pylori*-induced IL-8 production in gastric epithelial cells.	WA does not influence *H. pylori*-induced ROS production or any associated signaling.	[147]
*Withania somnifera* (Indian ginseng), Both aqueous as well as alcoholic extracts of the plant (root as well as leaves)	Pathogenic bacteria	In vitro study	Inhibitory activity against a spectrum of bacteria.	Increased survival rate as well as decreased bacterial load.	[148]
*Withania somnifera* (Indian ginseng) extracts	*Salmonella typhimurium* and *Escherichia coli*.	In vitro study	Methanol and hexane extracts of both leaves and roots were found to have potent antibacterial activity.	A synergistic increase in the antibacterial effect of Tibrim was noticed when MIC of Tibrim was supplemented with these extracts.	[149]
Extracts of *Withania somnifera* (Indian ginseng)	*Staphylococcus aureus*, *Escherichia coli*, *Pseudomonas aeruginosa* and *Bacillus subtilis*	In vitro study	Polar solvents had higher antibacterial property in comparison with the nonpolar solvents; higher MIC values were obtained for both gram-positive bacteria *S. aureus*, *B. subtilis* and gram-negative bacteria, *E. coli* and *P. aeruginosa*, with polar extract.	Antimicrobial activity of crude extract of *W. somnifera* was shown to validate the use of traditional medicinal herbal medicine and results of this study tend to give credence to the common use of *W. somnifera* plant.	[150]
*P. ginseng* polysaccharides	*H. pylori*	hemagglutination and enzyme-linked glycosorbent assays	Acidic carbohydrates may play an important role in the inhibitory activity on *H. pylori* adhesion to host cells.	Bacterial binding was inhibited more effectively by *P. ginseng* polysaccharides	[151]
Fermented ginseng extracts	*H. pylori*	Formation of clear zones, measurement of urease activity and cell adhesion activity in vitro.	Anti-*H. pylori* activity, including anti-bacterial, anti-adhesion, and urease inhibition effects.	Fermented ginseng extract containing *L.plantarum* MG 208 could prove to be useful as a functional diet for the protection of the gastric environment against *H. pylori.*	[152]
Red ginseng extracts (RGE)	*H. pylori*	Analysis of cell viability (trypan blue dye exclusion assay, DNA fragmentation assay (comet assay) Measurement of cytokine level, cell signaling (in vitro)	RGE decreased H. pylori-stimulated IL-8 gene expression, which resulted from the transcriptional regression of NF-κB.	RGE showed significant gastroprotective effects against *H. pylori*-associated gastric mucosal cell damage, suggesting that red ginseng could be used as a medicinal phytonutrient against *H. pylori* infection.	[153]
White ginseng extract (WGE)	*H. pylori*	Disc diffusion assay	The zone of inhibition due to WGE increased significantly with increasing dosage. WGE exhibited an inhibitory effect on cell growth at 2.0 mg/mL for all tumor cell lines.	The potential of WGE to be used as a health-promoting substance.	[154]
Ginseng aqueous extract	*Pseudomonas aeruginosa*	*P. aeruginosa* biofilms were further investigated in vitro and in vivo.	Oral administration of ginseng extracts in mice promoted phagocytosis of *P. aeruginosa* PAO1 by airway phagocytes but did not affect phagocytosis of a PAO1-film mutant.	Ginseng treatment may help to eradicate the biofilm-associated chronic infections caused by *P. aeruginosa.*	[155]
Saline extract of ginseng	*Pseudomonas aeruginosa*	Cytokine modulating effect in a mouse model of *P. aeruginosa* lung infection.	Th1-like immune response in the mice with *P. aeruginosa* lung infection after 7 days of ginseng treatment.	Th1 response might benefit the host with *P. aeruginosa* lung infection and ginseng treatment might be a promising alternative measure for the treatment of chronic *P. aeruginosa* lung infection in CF patients.	[156]
Polysaccharide (PS) isolated from *Panax ginseng*	*Staphylococcus aureus*	In vitro assays for the activity measurement of PS, NO production test with Greiss reagent, in vivo anti-septicemic activity was assessed by using C57BL/6J mice.	Polysaccharide showed anti-septic effects, Ginsan enhanced pro-inflammatory abilities (NO, pro-inflammatory cytokine production, phagocytic activity of macrophages). Ginsan modulated TLR pathway.	PS from *Panax ginseng* possess a potent anti-septicemic activity by stimulating macrophage and potential as an immunomodulator against sepsis caused by *Staphylococcus aureus.*	[157]
Polysaccharide (PS) isolated from *Panax ginseng*	*Staphylococcus aureus*	In vitro study	Proinflammatory cytokines, such as TNF-alpha, IL-1beta, IL-6, IFN-gamma, IL-12, and IL-18, were markedly down-regulated in ginsan-treated mice compared with those of control-infected mice.	Antiseptic activity of ginsan can be attributed to enhanced bacterial clearance, and reduced proinflammatory cytokines via the TLR signaling pathway.	[158]
Korean red ginseng	*Staphylococcus aureus*	Fluorescent marker calcein from negatively charged PC/PG (1: 1, *w*/*w*) liposomes	Ginsenosides may exert antibacterial activity by disrupting the cell membrane	Synergistic or additive effects between the ginsenosides and antibiotics tested	[159]
Crude saponins extracted from the *Panax quinquefolius*	*Fusobacterium nucleatum, Clostridium perfringens, and Porphyromonas gingivalis*	Determination of MIC, cell integrity	HTS, HTS-3, and HTS-4 were effective at inhibiting the growth *of F. nucleatum*, *C. perfringens*, and *P. gingivalis*.	Less polar ginsenoside-enriched fraction from heat transformation can be used as an antibacterial agent to control halitosis.	[160]
Acidic polysaccharide from *P. ginseng*, PG-F2	*P. gingivalis*	Determination of MIC	Anti-adhesive activity and anti-hemagglutination.	PG-F2 may exert a selective antiadhesive effect against pathogenic bacteria, while having no effects on beneficial and commensal bacteria.	[161]
A mixture of roasted coffee and red ginseng	*Pseudomonas aeruginosa* and *S. Typhimurium*	Classical paper disc method	DPPH scavenging activity decreased when red ginseng extract composed of more than 70% of the total extract.	Antibacterial activity shown.	[162]

Abbreviations: WA: Withaferin A; MIC: minimum inhibitory concentration; CF: cystic fibrosis; TLR: toll-like receptor; DPPH: 2,2 diphenyl-1-picryl-hydrazyl-hydrate; TNF-alpha: Tumor Necrosis Factor Alpha; ROS: reactive oxygen species; NF-Kb: Nuclear Factor kappa; MBC: minimum bactericidal concentration; KRG: Korean red ginseng; RGE: red ginseng extract; NO: nitric oxide; PC: Phosphatidylcholine; PG; Phosphatidyl glycerol; HTS: heat-transformed saponins; HTS-3 & HTS 4: Ginsenoside enriched fractions.

**Table 3:** Human trial findings to evaluate the effectiveness of ginseng against respiratory infections.

Participants	Interventions	Comparisons	Outcomes	Study Design	References
100 participants Age group 30–70 years	KRGE Nine capsules per day for three months	A Placebo-Controlled Trial of Korean Red Ginseng Extract to Prevent Acute Respiratory Illness in Healthy Subjects	Reduced Influenza-such as illness (ILI) incidence	Interventional (Clinical Trial)	[173]
43 participants ≥ 65 years of age	2 capsules/day of either COLD-fX or placebo (200 mg/capsule) for 4 months.	COLD-fX or placebo	Ingestion of COLD-fX by immunocompetent seniors during an early “cold and flu” season reduced the relative risk and duration of respiratory symptoms by 48% and 55%, respectively.	A randomized, double-blind, placebo-controlled trial.	[117]
783 community-dwelling adults.	Adults were randomized to receive placebo, 400 mg, or 800 mg treatment.	CVT-E002 (a proprietary) A double-blind, placebo-controlled trial.	CVT-E002 (a proprietary extract) can be safely used by similar groups and may prevent URI symptoms, Jackson-confirmed.	A multicenter, randomized, double-blind, placebo-controlled trial.	[174]
747 participants, more than equal to 18 years.	North American (*Panax quinquefolius*) or Asian ginseng (*Panax ginseng*) root extract or placebo or no treatment in healthy adults were included.	(*P. quinquefolius* or *P. ginseng*) root extract or placebo	Significantly reduced the total number of common colds by 25% compared with placebo. Tendency towards lower incidence of at least one common cold or other acute respiratory infection (ARI) in the ginseng group compared with the placebo group.	Randomized controlled trials or controlled clinical trials.	[175]
Eighty-nine (2000) and 109 (2000–2001) enrolled participants, average age 81 and 83.5, respectively; 74% women.	Oral twice-daily administration of a proprietary ginseng extract, CVT-E002, 200 mg, or placebo.	Proprietary extract of American ginseng, CVT-E002, with placebo in preventing acute respiratory illness (ARI)	CVT-E002 was shown to be safe, well tolerated, and potentially useful for preventing ARI due to influenza and RSV.	Two randomized, double-blind, placebo-controlled trials	[115]
323 subjects 18–65 years of age with a history of at least 2 colds in the previous year were recruited from Edmonton’s general population.	Two capsules per day of either the North American ginseng extract or a placebo for 4 months.	North American ginseng extract or a placebo.	Moderate dose over 4 months reduced the mean number of colds per person.	A randomized, double-blind, placebo-controlled	[116]
75 subjects, children 3 to 12 years of age.	Two dosing schedules of American ginseng extract during the winter months	American ginseng extract or a placebo.	Standard doses of ginseng were well tolerated and merit additional evaluation concerning pediatric upper respiratory tract infection treatment.	A randomized, double-blind dose-finding three-arm trial	[176]
14 participants (57–73 years old) with moderate to very severe COPD.	200 mg twice daily for four weeks) and then followed-up for an additional 4 weeks for a total of 10 weeks.	*P. ginseng* or a placebo	COPD exacerbations or adverse events	A randomized, double-blind, placebo-controlled clinical trial	[177]
500 children aged 3–11	Either COLD-FX or placebo for 3 days.	COLD-FX or a placebo	No results posted	A randomized, double-blind, placebo-controlled clinical trial.	ClinicalTrials.gov identifier (NCT number): NCT00965822
200 participants aged 12–75 years.	200 mg twice daily for 4 weeks. Other Name: CVT-E002	COLD-FX or a placebo	No results posted	A randomized, double-blind, placebo-controlled clinical trial.	ClinicalTrials.gov identifier (NCT number): NCT00726401
293 subjects with early-stage Chronic Lymphocytic Leukemia (CLL)	Oral extract for 3 months twice a day	COLD-FX or a placebo	Reduced rates of moderate-severe ARI and significantly less sore throat, enhanced antibody	A double-blind, placebo-controlled, randomized trial.	[178]
227 volunteers	Daily oral capsule doses of either placebo (113) or 100 mg of standardized ginseng extract Ginsana G 115 (114) for 12 weeks.	Ginsana G 115 (114) Or a placebo	Natural killer (NK) activity levels two-fold in the G115 group; can protect against common cold and influenza.	Multicenter, two-arm, randomized, placebo-controlled, double-blind	[81]
100 volunteers	Three times/day, 9 capsules/day, (3 g/day) for 12 weeks.	Korean Red Ginseng (KRG) or a placebo	May be useful in protecting subjects from contracting ARI and may decrease the duration and scores of ARI symptoms.	A randomized, double-blinded, placebo-controlled	[122]
45 healthy applicants aged 39–65 years	Six capules/day, 500 mg/capsule for 8 weeks.	GS-3K8 (ultra-filtered red ginseng extract) 2. GINst15 (hydrolyzed ginseng extract) or a placebo	GS-3K8 and GINST appear to have a positive tendency toward preventing ARI development and reducing the symptom duration.	A randomized, double-blind, placebo-controlled, pilot study at a single center.	[179]
328 subjects Age ≥ 18 Years	Sachet’s granules, Oral route at day 7	Drug: Jing Fang Bai Du san Drug: Placebo Drug: Ying Qiao san	Jing Fang Bai Du San relieved and effectively cleared up the pathogenic cold. Ying Qiao san effectively cleared up the pathogenic heat.	A randomized, double-blind, placebo-controlled	ClinicalTrials.gov identifier (NCT number): NCT00887172

Abbreviations: ARI: acute respiratory illness; CHM: Chinese herbal medicine; COPD: chronic obstructive pulmonary disease; LCCUs: laboratory-confirmed clinical upper respiratory infections; LCII: laboratory-confirmed influenza illness; NCT: clinical trial number; URTI: upper respiratory tract infection; KRGE: Korean Red Ginseng extract; RSV: respiratory syncytial virus.
